# Degradable Dextran Nanopolymer as a Carrier for Choline Kinase (ChoK) siRNA Cancer Therapy

**DOI:** 10.3390/nano6020034

**Published:** 2016-02-22

**Authors:** Zhihang Chen, Balaji Krishnamachary, Zaver M. Bhujwalla

**Affiliations:** JHU ICMIC Program, Division of Cancer Imaging Research, Russell H. Morgan Department of Radiology and Radiological Science, The Johns Hopkins University School of Medicine, Baltimore, MD 21205, USA; zchen19@jhu.edu (Z.C.); bkrishn1@jhmi.edu (B.K.)

**Keywords:** siRNA therapy, choline kinase, dextran, nano-polymer, siRNA delivery

## Abstract

Although small interfering RNA (siRNA) therapy has proven to be a specific and effective treatment in cells, the delivery of siRNA is a challenge for the applications of siRNA therapy. We present a degradable dextran with amine groups as an siRNA nano-carrier. In our nano-carrier, the amine groups are conjugated to the dextran platform through the acetal bonds, which are acid sensitive. Therefore this siRNA carrier is stable in neutral and basic conditions, while the amine groups can be cleaved and released from dextran platform under weak acid conditions (such as in endosomes). The cleavage and release of amine groups can reduce the toxicity of cationic polymer and enhance the transfection efficiency. We successfully applied this nano-carrier to deliver choline kinase (ChoK) siRNA for ChoK inhibition in cells.

## 1. Introduction

Since the first application of small interfering RNA (siRNA) in gene knockdown, this technology has rapidly become a powerful tool in basic research [1]. The technology is being actively investigated for molecular-based therapeutic strategies in several diseases including cancer [2,3], inflammation [4], diabetes [5], and neurodegenerative diseases [6,7]. siRNA can enter the RNA-induced silencing complex (RISC), which induces enzyme-catalyzed degradation of their complementary messenger RNAs (mRNAs) in diseased cells, thus disrupting specific molecular pathways in various diseases [8]. Therefore siRNA has garnered considerable interest as a specific, safe, and potent therapeutic agent. However naked siRNA does not readily cross the anionic cell membrane through passive diffusion [9,10] because of its net negative charges on the phosphate backbone large size, and the high molecular weight. Hence the development of a safe and efficient siRNA delivery system still remains a critical challenge.

For the efficiency delivery of siRNA, a large number of carriers, such as viral vectors [11], cationic lipids [12,13], and polymers [14,15] have been explored. Despite high transfection capability, the applications of viral carriers are limited by serious immunotoxicity, mutagenicity and oncogenesis [9]. Therefore cationic polymers have attracted attention because of low cost, easy synthesis and modification. Cationic polymers can efficiently assemble with siRNA through electrostatic interaction, and this positively charged condensed complex can enhance cellular uptake and siRNA-mediated gene-silencing [10]. However, such complexes with high positive surface charge often induce an inflammatory response that causes severe toxicities *in vivo* and *in vitro* [16,17]. Therefore it is important to develop a safe and efficient carrier for siRNA delivery.

Here we present an imaging reporter labeled dextran-based biodegradable nano-polymer based on dextran as a safe carrier for choline kinase (ChoK) siRNA cancer therapy. ChoK overexpression and increased activity have been observed in malignant cells and tumors of the lung, colon, breast, prostate, and ovaries [18,19]. Therefore ChoK is an excellent target for cancer gene therapy, and ChoK siRNA therapy has been investigated for cancer treatment in preclinical models [20,21]. Dextran has been used as a polymeric carrier because of its wide availability, biodegradability, and ease of modification [22]. The potential application of dextran for siRNA delivery has recently been demonstrated [23,24,25,26]. Amine function groups, which provide the positive charge to bind with siRNA through electrostatical interaction, were conjugated to the dextran platform through acetal bonds. Acetal bonds are attractive because of the breakage of the bond under acidic conditions, which exist at various diseased sites such as tumors and inflammation, as well as inside endocytic compartments [27]. Therefore the amine groups can be cleaved from the dextran backbone and are rapidly released from the cells. The elimination of amine groups can minimize the inflammatory response and the toxicity of cationic dextran siRNA carriers. The labeled imaging probe provides the potential to use the nano-carrier as a fluorescent theranostic nanoplex.

## 2. Results and Discussion

**Scheme 1:**
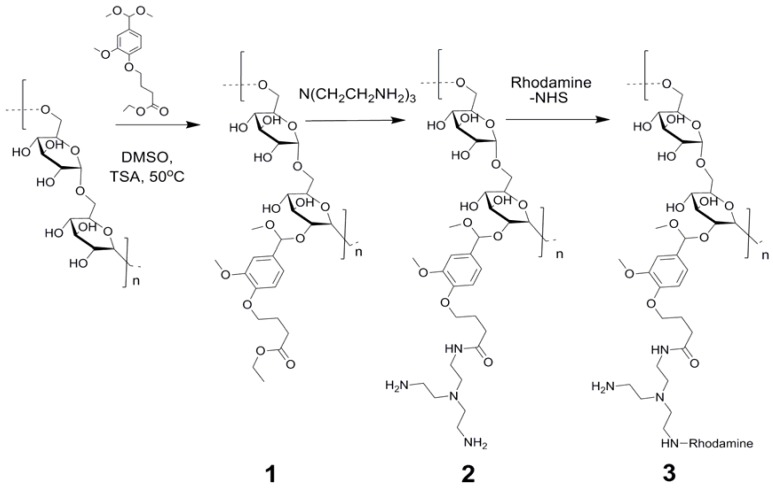
Synthetic procedure of generating a degradable amino-dextran small interfering RNA (siRNA) carrier. **1**: Dextran with acetal group; **2**: Amino-dextran; **3**: Rhodamine labeled amino-dextran.

The synthesis of the dextran polymer is presented in Scheme 1. At first, dextran (70 kDa) was reacted with an overdose of ethyl 4-(formyl-3-methoxyl)phenyl butyrate dimethyl acetal to produce the dextran compound **1** with acetal bonds. Amine groups were introduced to the dextran by the reaction between the ester group of dextran and tris(2-aminoethyl)amine to form the dextran compound **2**. ^1^H NMR spectra confirmed that approximately 69% of the glucose residues were functionalized. Finally the rhodamine NHS ester reacted with these amine groups to form the rhodamine (1.2 rhodamine molecule per dextran molecule) labeled dextran siRNA carrier (compound **3**).

**Figure 1:**
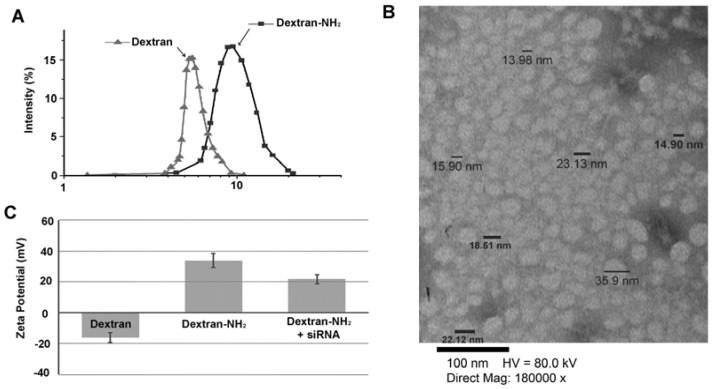
(**A**) Hydrodynamic radius of dextran and amino-dextran dextran from intensity-based distributions. (**B**) Transmission electron microscopy (TEM) image of amino-dextran. Negative staining with phosphotungstic acid (PTA), scale bar is 100 nm. (**C**) Zeta potential of dextran and amino-dextran dextran. *n* = 3, values represent Mean ± standard deviation (SD).

The hydrodynamic radius of the dextran carriers was investigated by dynamic light scattering (DLS), and the result is shown in Figure 1A. The radius of natural dextran (70 kDa) was around 6 nm, and the radius increased to around 9.95 nm (intensity-based distributions) after modifications. Since this nano-carrier is a polydisperse polymer (polydispersity index (DPI) of DLS is 0.36), the peak of number-based size distributions is only 7.90 nm (Figure S1). TEM images (a representative image is shown in Figure 1B) indicated that the diameters of these degradable amino-dextran nano-carriers were in the range of 13 nm and 35 nm, and this result matched the result obtained with DLS. Due to the negative zeta potential (−16.12 ± 3.25 mV) (Figure 1C), natural dextran cannot bind with siRNA efficiently. In our degradable amino-dextran siRNA nano-carrier, the induced cleavable amine groups increased the zeta potential of dextran to 33.84 ± 4.5 mV; therefore this amino-dextran (compound **3**) can provide efficient binding of the cargo to the carrier for successful gene delivery. Gel electrophoresis was used to examine the complexation between siRNA and the amino-dextrans. The amino-dextrans formed firm complexes with siRNA when nitrogen/phosphate (N/P) ratios were over 10, and the zeta potential of dextran/siRNA complexes at N/P ratio of 15 was 21.83 ± 2.94 mV.

**Figure 2:**
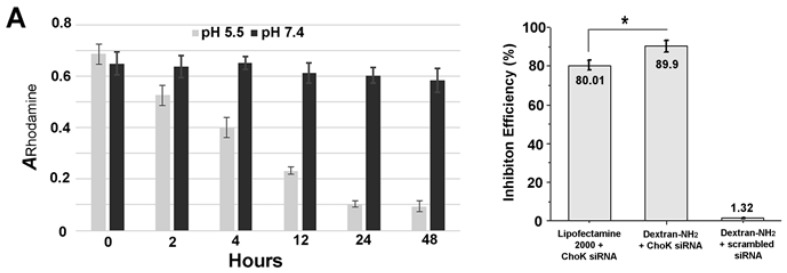
(**A**) The rhodamine absorbance of 1 mg/mL dextran solution in pH 5.5 and pH 7.4 buffer at different time points. *n* = 3, values represent Mean ± SD. (**B**) Inhibition efficiency of choline kinase (ChoK) messenger RNA (mRNA) in MDA-MB-231 cells with different siRNA transfection agent treatments. Small interfering RNA (siRNA) concentration: 100 nM; N/P ratio: 15. Cells were treated with siRNA/dextran for 24 h, following by a further 6 h incubation in fresh medium. *n* = 3, values represent Mean ± SD. *, *p* < 0.05.

In molecular reagent (nucleic acid, siRNA, *etc.*) therapy, the efficient release of the molecular reagent from the carrier is important to achieve high transfection efficiency. In our degradable amino-dextran nano-carrier, the acetal bonds, which linked amine groups to the dextran platform, were easily broken under acidic conditions, such as in endosomes, and this breakage of acetal bonds induced cleavage of the amine group. The elimination of the amine functional group reduced the positive charge of the dextran delivery system, and the siRNA with negative charge was released quickly. Here our degradation studies of amino-dextran were performed by using colorimetric assay of rhodamine in different pH buffers (acetate buffer, pH 5.0 and phosphate-buffered saline (PBS) buffer, pH 7.4). After incubation in buffer, the amino-dextran nano-carrier was purified by molecular weight cut-off centrifugation and lyophilization. The progress of degradation was monitored by comparing the absorbance of rhodamine of dextran solution at a specific concentration (1 mg/mL) at 530 nm. Rhodamine conjugated to the amine group is cleaved under weak acid condition, therefore the absorbance of rhodamine in the dextran carrier decreased with time in a pH 5.0 PBS buffer. The acetal bond, however, degraded very slowly at neutral and basic conditions, as a result of which the absorbance of rhodamine did not show a significant change in neutral and basic solutions. As shown in Figure 2A, in PBS (pH 7.4) buffer, there was no obvious degradation, and the absorbance of rhodamine was around 0.6. In contrast, the amino-dextran carrier degraded and released the small molecule containing amine rapidly at pH 5.5. After 2 h incubation, the absorbance of rhodamine decreased from 0.68 to 0.52, and this decrease of absorbance of rhodamine was increasingly evident as the incubation time proceeded. These compounds reached nearly complete degradation (over 85%) after 48 h incubation in pH 5.0 buffer. The MTT assay was applied to evaluate the cytotoxicity of our dextran-NH_2_ nano-carrier. , Cytotoxicity was not observed following a 72 h treatment with 0.5 mg/mL of the dextran nano-carrier. We also investigated the transfection efficiency of our degradable amino-dextran nano-carrier *in vitro*. Quantitative reverse transcription polymerase chain reaction (qRT-PCR) was performed to measure ChoK mRNA level in the triple negative MDA-MB-231 breast cancer cell line. Cells were treated with ChoK siRNA for 24 h, and the medium containing the released amine was replaced with fresh medium. With 6 h of further incubation in fresh medium, cells were collected for qRT-PCR assay. As shown in Figure 2B, when lipofectamine 2000, which is a commercial transfection agent, was applied as an siRNA carrier, the inhibition efficiency was 80.01%. In contrast, our degradable amino-dextran nano-carrier demonstrated a better inhibition efficiency (87.9%) than lipofactamine 2000. Treatment of cells with the dextran-NH_2_ nano carrier carrying scrambled siRNA, as the negative control, did not induce ChoK inhibition.

**Figure 3:**
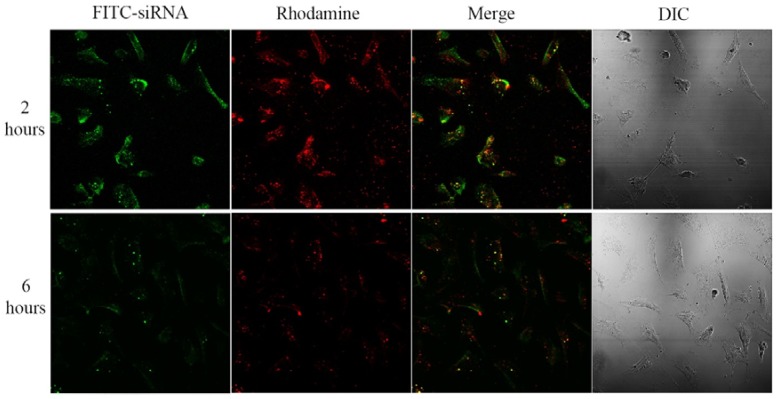
Representative laser confocal fluorescence microscopy fields of view of MDA-MB-231 cells treated with siRNA/dextran nano-carrier. Cells were treated with fluorescein isothiocyanate (FITC)-siRNA/dextran nano-carrier at a concentration of siRNA: 50 nM and N/P ratio: 15.

Investigation of the distribution and degradation of amino-dextran in cells was performed with a laser scanning confocal microscope (Figure 3). In general, non-viral delivery systems enter cells through the endocytic vesicles that are weakly acidic. When degradable amino-dextran/siRNA nano-carriers are taken up in cells through these endocytic vesicles, the amine groups, which are conjugated to the dextran platform through acid sensitive acetal bonds, are cleaved in the endosomes. In our experiments, the cells were treated with fluorescein isothiocyanate (FITC) labeled siRNA/dextran for 1 h, following which cells were further incubated in fresh medium. In Figure 3, the fluorescence of FITC is shown in green, and the fluorescence of rhodamine is shown in red. After 2 h further incubation, strong fluorescence from FITC and rhodamine was observed. Although the co-localization of the fluorescence from FITC and rhodamine was dominant, delocalized fluorescence of FITC and rhodamine was also observed indicating partly released siRNA. At 6 h after treatment, the fluorescence from FITC and rhodamine was still detected, but these were much weaker than the fluorescence at 2 h incubation indicating that most of acetal bonds were cleaved, and the siRNA was released almost completely. Investigation of the role of the endocytosis pathway in the uptake of the nano-carriers was performed by analyzing the fluorescence intensity of the endocytosed dextran nan-carriers in the presence of various endocytosis inhibitors. The relative intensities of red fluorescence (normalized to intensity of no inhibitor treatment) are listed in Table 1. Cytochalasin D was used to inhibit phagocytosis and micropinocytosis [28]; chlorpromazine hydrochloride was used to inhibit clathrin-mediated endocytosis [29]; methyl-β-cyclodextrin was used to inhibit caveolae mediated endocytosis [30]; nocodazole was used as a microtubule-disrupting agent [31]. In these treatments, chlorpromazine hydrochloride, methyl-β-cyclodextrin and nocodazole demonstrated inhibition of the nano-carrier uptake. Weak uptake inhibition by cytochalasin D indicated that the uptake of dextran compound is less dependent on phagocytosis and micropinocytosis. The significant reduction of fluorescence intensity of dextran at low temperature indicates an energy dependent uptake.

## 3. Experimental Section

### 3.1. Determination of Size Distribution and Zeta Potential

The hydrodynamic radius and size distribution degradable amino-dextran nano-carrier (compound **3**) and natural dextran (70 kDa) were determined by dynamic light scattering (DLS, 10 mW He-Ne laser, 633 nm wavelength, (Malvern, Westborough, MA, USA). The DLS measurements were performed in triplicate. Dextran-siRNA nanocomplex was prepared at N/P ratios of 15 by adding a PBS buffer solution (20 mM, pH 7.4) of compound **3** (600 μL) to a distilled water solution of siRNA (400 μL, 50 μg/mL), followed by vortexing for 5 s and incubating for 10 min at room temperature. The average zeta potential of natural dextran, compound **3** (amino-dextran) and dextran-siRNA nanoplex (N/P = 15) in ddH_2_O were measured with a Zetasizer Nano ZS instrument (Malvern, Westborough, MA, USA) equipped with a clear standard zeta capillary electrophoresis cell cuvette from 20 acquisitions with a concentration of approximately 0.5 mg/mL. All measurements were performed in triplicate.

### 3.2. Colorimetric Assay of Degradation Study

10 mg of compound **3** was dissolved in the desired pH value buffer, and incubated at 37 °C. After incubation, the solution was transferred into 15 mL molecular cut-off centrifugal filters (EMD Millipore, Billerica, MA, USA) (MW 10 kDa) and spun at 3000 g for 15 min to remove the small molecules. It was washed with deionized water in this molecular cut-off centrifugal filter for 2 more times. Then it was frozen in liquid nitrogen, and lyophilized. After lyophilization, the dextran powder was dissolved in pH 7.4 PBS buffer to form 1 mg/mL dextran solution, and the absorbance at 530 nm was measured.

### 3.3. Cell Culture and RNA Isolation, Quantitative Reverse Transcription-PCR (qRT-PCR)

Human breast cancer MDA-MB-231 cells were obtained from American Type Culture Collection (ATCC) (Manassas, VA, USA). Fetal bovine serum, penicillin, and streptomycin were from Invitrogen (Carlsbad, CA, USA). Cells were maintained in RPMI 1640 (Invitrogen, Grand Island, NY, USA) supplemented with 10% fetal bovine serum in a humidified incubator at 37 °C/5% CO_2_. Cells were seeded at a density of 400,000 cells per dish in 6 cm dish (for RT-PCR experiments) or 8000 cells per well in 8 wells slide chamber (for confocal laser scanning fluorescence microscopy studies) 24 h prior to the transfection experiment. The siRNA/dextran RPMI 1640 medium solution (concentration of siRNA: 100 pmol/mL, N/P = 15) was added to each well or dish. Total RNA was isolated from MDA-MB-231 cells grown in 60mm dish by using QIAshredder and RNeasy Mini kit (Qiagen, Valencia, CA, USA) as per the manufacturer’s protocol. The expression of target RNA relative to the housekeeping gene HPRT1 was calculated based on the threshold cycle (Ct) as *R* = 2 − Δ(ΔCt), where ΔCt = Ct of target − Ct of HPRT1. The following primers against (a) ChoK-Fwd-5’-GAAAGTGCTCCTGCGGCTGTATG-3' and Rev-5’-CGGCTCGGGATGAACTGCTC-3', (b) HPRT1-the house keeping gene-Fwd-5'-CCTGGCGTCGTGATTAGTGATG-3' and Rev-5'-CAGAGGGCTACAATGTGATGGC-3' were designed using either Beacon designer software 7.8 (Premier Biosoft, Palo Alto, CA, USA) or a free web-based software Primer3Plus software (Premier Biosoft, Palo Alto, CA, USA) [32].

### 3.4. Confocal Laser Scanning Fluorescence Microscopy

MDA-MB-231 cells in 8 well chamber slides were treated with siRNA/compound **3** nanoplex (concentration of siRNA: 100 pmol/mL, N/P = 15) for 1 h. After incubation, the transfection mixture was removed, and cells were washed twice with fresh medium. Cells were incubated in fresh medium for further observation. In endocytosis experiments, the cells were treated with endocytosis inhibitors for 45 min at 37 °C/5% CO_2_, following which the dextran nano-carrier was added into medium for a further 2 h incubation at 37 °C. Fluorescence microscopy images of MDA-MB-231 cells were generated on a Zeiss LSM 700 META confocal laser-scanning microscope (Carl Zeiss, Inc., Oberkochen, Germany).

### 3.5. Statistical Analysis

Values are presented as mean ± standard deviation. Statistical differences were evaluated with Student’s t test (Excel 2015, Microsoft, Redmond, WA, USA), *p* < 0.05 (two tailed) was considered significant.

## 4. Conclusions

We synthesized a degradable dextran as an siRNA nano-carrier. In this degradable dextran nano-carrier, amine groups were conjugated to a dextran platform through acid sensitive acetal bonds. These amine groups could be cleaved under weak acid condition, but were stable at weak basic condition (such as in pH 7.4 buffer). The cleavage and release of amine groups can improve the safety of cationic dextran siRNA nano-carrriers, and accelerate the release of siRNA to enhance the transfection efficiency. Our experiments demonstrated that this degradable dextran nano-carrier delivered ChoK siRNA efficiently, and inhibited ChoK expression in MDA-MB-231 cells. Cell imaging proved this compound degraded rapidly, and delivered and released siRNA efficiently. This degradable dextran nano-carrier should be further evaluated for applications as a safe, reproducible, and biocompatible siRNA carrier for siRNA therapy in preclinical studies.

## Figures and Tables

**Table 1:** Relative fluorescence intensities of cellular up-taken dextran carrier with endocytosis inhibitors.

Treatment	Relative fluorescence intensities
No inhibitor	1 ± 0.0484
Cytochalasin D (5 µg/mL)	0.91 ± 0.0678
Chlorpromazine hydrochloride (10 µg/mL)	0.45 ± 0.0453
Methyl-β-cyclodextrin (5 mM, 6.5 mg/mL)	0.57 ± 0.0261
Nocodazole (20 µM)	0.52 ± 0.0148
Low temperature (4 °C)	0.29 ± 0.0650

## Supplementary Materials

The following are available online at http://www.mdpi.com/2079-4991/6/2/34/s1.
