# Supplementary material for: Degradable Dextran Nanopolymer as a Carrier for Choline Kinase (ChoK) siRNA Cancer Therapy
Source: Nanomaterials (Basel). 2016 Feb 22;6(2):34. doi: 10.3390/nano6020034 (PMC5302479; doi:10.3390/nano6020034)
Supplement: Supplementary file 1 [file nanomaterials-06-00034-s001.pdf]

Supplementary Materials: Degradable Dextran Nanopolymer as a Carrier for Choline Kinase (ChoK) siRNA Cancer Therapy

Zhihang Chen, Balaji Krishnamachary and Zaver M. Bhujwalla

1. Materials and General Experimental Methods

All organic chemicals and solvents were of analytical grade and purchased from Aldrich (St. Louis, MO, USA), Sigma (Milwaukee, WI, USA) and Alfa Aesar (Ward Hill, MA, USA) unless otherwise specified. Fetal bovine serum (FBS), penicillin, and streptomycin were obtained from Invitrogen (Carlsbad, CA, USA). Amicon ultra-15 centrifugal filter tubes (10,000 MW cutoff) were procured from Millipore (Bedford, MA, USA). The triple negative human breast cancer cell line MDA-MB-231 was purchased from American Type Culture Collection (Rockville, MD, USA). ^{1}H, ^{13}C NMR spectra were recorded at 400 MHz on Varian Mercury400 spectrometer (Varian Inc., Palo Alto, CA, USA), and chemical shifts were reported in ppm relative to tetramethylsilane (TMS).

2. Synthesis Procedure

Compound 1: 30.0 mg dextran (70 KDa) was dissolved in anhydrous DMSO (1 mL), followed by addition of dimethyl acetal (ethyl 4-(4-formyl-3-methoxy)phenyl butyrate dimethyl acetal, [1] 350.0 mg), molecular sieves (5 Å, 30 mg), and p-toluenesulfonic acid monohydrate (3.0 mg). The reaction mixture was heated for overnight at 80 °C, and the reaction was quenched by addition of triethylamine. Molecular sieves were then filtered, and the DMSO solution was dripped into a mixture of isopropyl alcohol or isopropyl alcohol and hexanes (1:1) to obtain white precipitate. This white precipitate was purified through washing with isopropyl alcohol and hexane. After drying, 68.5 mg water-insoluble white powder was obtained. Due to the rather complex and uninterpretable ^{1}H NMR spectrum in d6-DMSO, characterization was performed at the following step to confirm the structure of compound **1**.

Compound 2: 40.0 mg compound **1** was dissolved in 2 mL anhydrous DMSO, followed by addition of tris(2-aminoethyl)amine (150 μL). The mixture was kept at 50 °C for two weeks. After reaction, the DMSO solution was dropped into ethyl acetate to precipitate the white product. The suspension was centrifuged to obtain the white precipitate, which was purified by three reprecipitations in ethyl acetate. After drying, 39.8 mg of white product (yield: 89%) was obtained. ^{1}H NMR (500 MHz, D_2O) δ 7.24–6.75 (m. (multiplet), 0.69 × 4H, Ar), 6.12–5.43 (m, 0.69 × 1H, acetal-H), 5.38–4.92 (m, 1H, glucose-H_1), 4.37–3.32 (m, 6H, glucose-H_{2-6}; 0.69 × 2H, ArOCH_2; 0.69 × 3H, ArOCH_3; 0.69 × 2H, CH_2NHCO; 0.69 × 3H, acetal-OCH_3), 2.61–2.83 (br. app. s. (broad apparent singlet), 0.69 × 4H, NHCH_2CH_2NH_2; 0.69 × 4H, NHCH_2CH_2NH_2; 0.69 × 2H, CONHCH_2CH_2NH), 2.48 (br. app. s, 0.69 × 2H, CH_2CO), 2.08 (br. app. s, 0.69 × 2H, ArOCH_2CH_2).

Compound 3: 30.0 mg compound **2** was dissolved in 2 mL HEPES buffer (pH 8.4), then 0.5 mg rhodamine 6G NHS ester in 100 μL DMSO was added into the dextran solution. The mixture was stirred at room temperature for 2 hours, and transferred into centrifugal filter tubes (Amicon ultra-15, 10,000 MW cutoff) for purification. After 3 times purification and lyophilization, 27.1 mg of the purple solid was harvested. Colorimetric assay of rhodamine 6G at 530 nm indicated that there were 1.2 rhodamine molecules in one dextran molecule. NMR could not characterize conjugation of rhodamine due to the very low concentration of rhodamine.

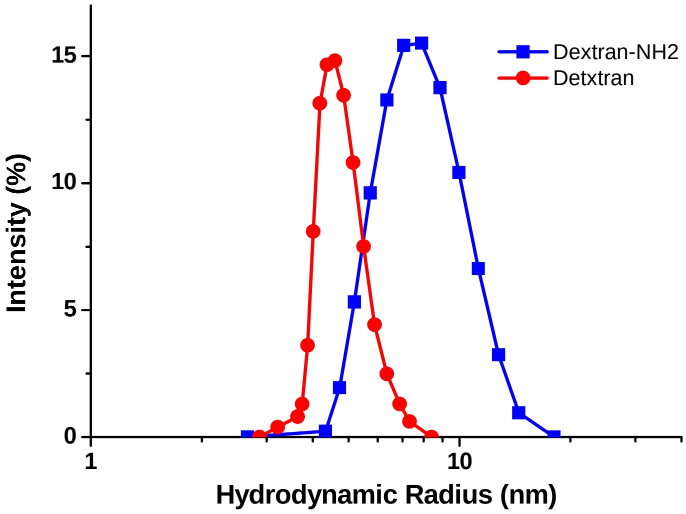

Figure S1. Hydrodynamic radius of dextran and amino-dextran dextran (number-based distributions).

Reference

1. Cui, L.; Cohen, J.L.; Chu, C.K.; Wich, P.R.; Kierstead, P.H.; Fréchet, J.M.J. Conjugation Chemistry through Acetals toward a Dextran-Based Delivery System for Controlled Release of siRNA. *J. Am. Chem. Soc.* **2012**, *134*, 15840–15848.

© 2016 by the authors; licensee MDPI, Basel, Switzerland. This article is an open access article distributed under the terms and conditions of the Creative Commons by Attribution (CC-BY) license (http://creativecommons.org/licenses/by/4.0/).
